# Supplementary material for: Comparison of early effects of pneumococcal conjugate vaccines: PCV7, PCV10 and PCV13 on Streptococcus pneumoniae nasopharyngeal carriage in a population based study; The Palestinian-Israeli Collaborative Research (PICR)
Source: PLoS One. 2018 Nov 12;13(11):e0206927. doi: 10.1371/journal.pone.0206927 (PMC6231627; doi:10.1371/journal.pone.0206927)
Supplement: S1 Table — (DOCX) [file pone.0206927.s001.docx]

**S1 Table**: Characteristics of the two study populations in each of the four screening periods.

|  | **PA** | | | **EJ** | | | | |  |  | |  |
| --- | --- | --- | --- | --- | --- | --- | --- | --- | --- | --- | --- | --- |
| **2014** | **2011** | **2010** | **2009** |  | **2014** | **2011** | **2010** | **2009** |  |  | |  |
| 643 | 555 | 595 | 620 |  | 287 | 324 | 311 | 345 |  | Number of children | |  |
| 380 (59.1%) | 346 (62.6%) | 376 (64.0%) | 378 (60.9%) |  | 154 (53.7% | 176 (54.7%) | 173 (55.6%) | 208  (59.8%) | Male  N (%) | | Sex | |
| 149 (23.2%) | 175 (31.5%) | 195 (32.9%) | 140 (22.5%) |  | 32 (11.2%) | 42  (13.0%) | 59 (19.0%) | 47  (13.5%) | <6 | | Age  (in months) | |
| 298 (46.4%) | 243 (43.6%) | 249 (42.1%) | 294 (47.4%) |  | 143 (49.8%) | 154 (47.5%) | 128 (41.1%) | 122  (35.1%) | 6-23 | |  | |
| 196 (30.5%) | 137 (24.6%) | 148 (25.0%) | 186 (29.9%) |  | 112 (39.0%) | 128 (39.5%) | 124 (39.9%) | 179  (51.4%) | 24-60 | |  | |
| 225 (35.0%) | 187 (33.7%) | 185 (31.4%) | 143 (23.0%) |  | 75 (26.7%) | 56 (17.3%) | 84 (27.0%) | 75  (21.6%) | <4 | | Number of household members | |
| 321 (49.9%) | 261 (46.9%) | 308 (52.5%) | 326 (52.5%) |  | 158 (56.2%) | 192 (59.4%) | 173 (55.6%) | 207  (59.5%) | 4-6 | |  | |
| 97 (15.1%) | 107 (19.2%) | 97 (16.4%) | 152 (24.5%) |  | 48 (17.1%) | 75 (23.2%) | 54 (17.4%) | 66  (19.0%) | >6 | |  | |
| 254 (39.5%) | 269 (48.3%) | 263 (44.2%) | 348 (56.0%) |  | 132  (46.0%) | 197 (60.8%) | 153 (49.2%) | 187  (53.7%) | Received recent antibiotics (last 3 months) | | | |
